# Novel multifunctional robotically assisted bipolar instrument for simultaneous radiofrequency sealing and transection: preclinical and single-center experience

**DOI:** 10.1186/s12893-022-01483-5

**Published:** 2022-02-02

**Authors:** Cristina Ibanez Jimenez, Adrit Lath, Forrest Ringold

**Affiliations:** 1 Clinical Development Engineering, Intuitive Surgical, Inc., Sunnyvale, CA USA; 2 New Product Development, Advanced Energy Instruments, Intuitive Surgical, Inc., Sunnyvale, CA USA; 3 Surgical Association of Mobile, Mobile, AL USA; 4 Mobile Infirmary Medical Center, Mobile, AL USA; 5 Springhill Medical Center, Mobile, AL USA

**Keywords:** Vessel sealing, Transection, Safety, Robotic assistance, Radiofrequency energy, Ultrasonic energy

## Abstract

**Background:**

A novel robotic-assisted bipolar radiofrequency (RF) multifunctional vessel seal-and-transection instrument (SynchroSeal, Intuitive Surgical, Inc., Sunnyvale, CA) has been developed. The objective of the current paper is to describe the design of SynchroSeal based on bench studies, assess the safety of SynchroSeal in ex vivo and in vivo porcine studies, and provide early clinical context.

**Methods:**

SynchroSeal grasping, energy activation time, and jaw temperature were evaluated with those of the Harmonic Ace+7. Data were analyzed with descriptive statistics, with Mann–Whitney for comparisons and statistical significance *p *< 0.05. Ex vivo and in vivo animal safety assessments of tissue after SynchroSeal use were evaluated for burst pressure, thermal spread, and acute sealing. Last, a single-center analysis of the technical metrics of SynchroSeal and Vessel Sealer Extend (robotically assisted seal-and-transection instrument) in bariatric cases is provided.

**Results:**

Bench studies of SynchroSeal and Harmonic Ace+7 evidenced SynchroSeal’s greater slip resistance force (8.4 ± 1.0 vs. 3.1 ± 0.4 N; *p* = 0.0002), lower grip pressure (3.0 ± 0.2 vs. 4.2 ± 0.5 kg/cm^2^; *p* = 0.0002), faster seal time (1.5 ± 0.4 vs. 11.6 ± 2.5 s; *p *< 0.0001), lower mean jaw temperature (109.7 ± 7.2 vs. 247.4 ± 8.6 °C; *p* = 0.0051), and faster cooling to 40 °C (53.6 ± 2.1 vs. 68.0 ± 3.5 s; *p* = 0.0051). SynchroSeal’s mean burst pressures after seal-and-transection and seal only modes were, respectively, 1169.1 ± 393.1 mmHg and 1159.2 ± 454.6 mmHg. Mean thermal spreads were, respectively, 1.2 ± 0.6 mm and 1.5 ± 0.55 mm. In the chronic animal study, 102 vessels were sealed; at 3 weeks post-procedure, there was no evidence of leakage or adverse events, such as non-target tissue thermal spread or tissue damage. In bariatrics cases, SynchroSeal was activated more frequently per case; however, its mean activation time was significantly shorter than Vessel Sealer Extend. No adverse events were reported for either device.

**Conclusions:**

SynchroSeal’s multifunctional design provides enhanced sealing and transection capabilities with an acceptable safety profile.

## Background

The ability to seal and transect vessels and tissue effectively and safely is a critical surgical task. Different energy delivery methods like radiofrequency (RF) and ultrasonic energy have been successfully implemented to produce effective vessel sealing and cutting instruments. With added focus on surgical efficiency, hospitals and surgeons prefer more multifunctional instruments to reduce the number of different surgical instruments as well as using ones that reduce procedure times with no impact on safety. Established technology has involved clamping of vessels with controlled pressure and application of RF energy to heat, coagulate, and fuse the tissue, thereby sealing the vessel at the energy application site [1]. Computer algorithms monitor tissue impedance, thereby controlling the applied energy, and turn off the RF energy at the optimal time for successful vessel sealing [2].

Sealing and transection instruments based on bipolar, ultrasonic and hybrid energy have been developed [3–5]. A novel bipolar instrument, SynchroSeal® (Intuitive Surgical, Inc., Sunnyvale, CA USA), has been developed for the da Vinci Surgical Systems (Intuitive Surgical, Inc., Sunnyvale, CA USA) and incorporates fully wristed seal and RF transection capabilities to improve precision and access and to minimize potential damage to surrounding tissue [6]. The novelty of SynchroSeal lies in its ability to transect using RF energy, which allows for a single step seal-and-cut, differentiating it from other bipolar vessel sealers. SynchroSeal is fully integrated with the robotic-assisted system: tissue clamping pressure as well as sensing and messaging of seal success or errors are controlled and communicated by the robotic system.

The objective of the current study is to validate the novel RF sealing and RF transection design of SynchroSeal based on comparative bench studies with the ultrasonic energy-based Harmonic® Ace®+7 (Ethicon Endo-Surgery Inc., Cincinnati, OH USA) instrument, given that SynchroSeal shares similar features to Harmonic Ace+7, such as the jaw shape and single step seal-and-transection, and to assess SynchroSeal in an ex vivo and an in vivo animal safety study and provide early clinical context for the preclinical findings.

## Methods

Bench studies of the SynchroSeal were carried out to validate its design as a multifunctional seal and transection device. Its grasping, energy activation time, and jaw temperature profiles were compared with those of the Harmonic Ace+7, a single-use ultrasonic instrument used for ultrasonic coagulation and ultrasonic transection of soft tissue and vessels up to 7 mm in diameter during laparoscopic and open procedures.

In addition to the bench studies, ex vivo and in vivo animal safety assessments of SynchroSeal were made to evaluate treated tissue for burst pressure, thermal spread, and acute seal. Sample sizes were determined based on data from a pilot study.

In addition, a single user, single center retrospective comparison of technical and safety outcomes from use of SynchroSeal (E-100 electrosurgical generator; Intuitive Surgical Inc., Sunnyvale, CA) and an earlier robotic-assisted instrument, Vessel Sealer Extend (VSE) (ERBE VIO dV generator; Erbe Elektromedizin GmbH, Tübingen Germany), in consecutive cases of sleeve gastrectomy and Roux-en-Y gastric bypass is presented to provide clinical context for the technical data. VSE is a bipolar, electrical vessel sealing instrument for use on the da Vinci platform. It can seal vessels ≤ 7 mm in diameter and tissue bundles that fit within the tool’s jaws. Transection is accomplished with a mechanical knife that traverses the jaws. The VSE jaws are straight and taper from 5.1 mm proximally to 3.2 mm distally.

**Fig. 1:**
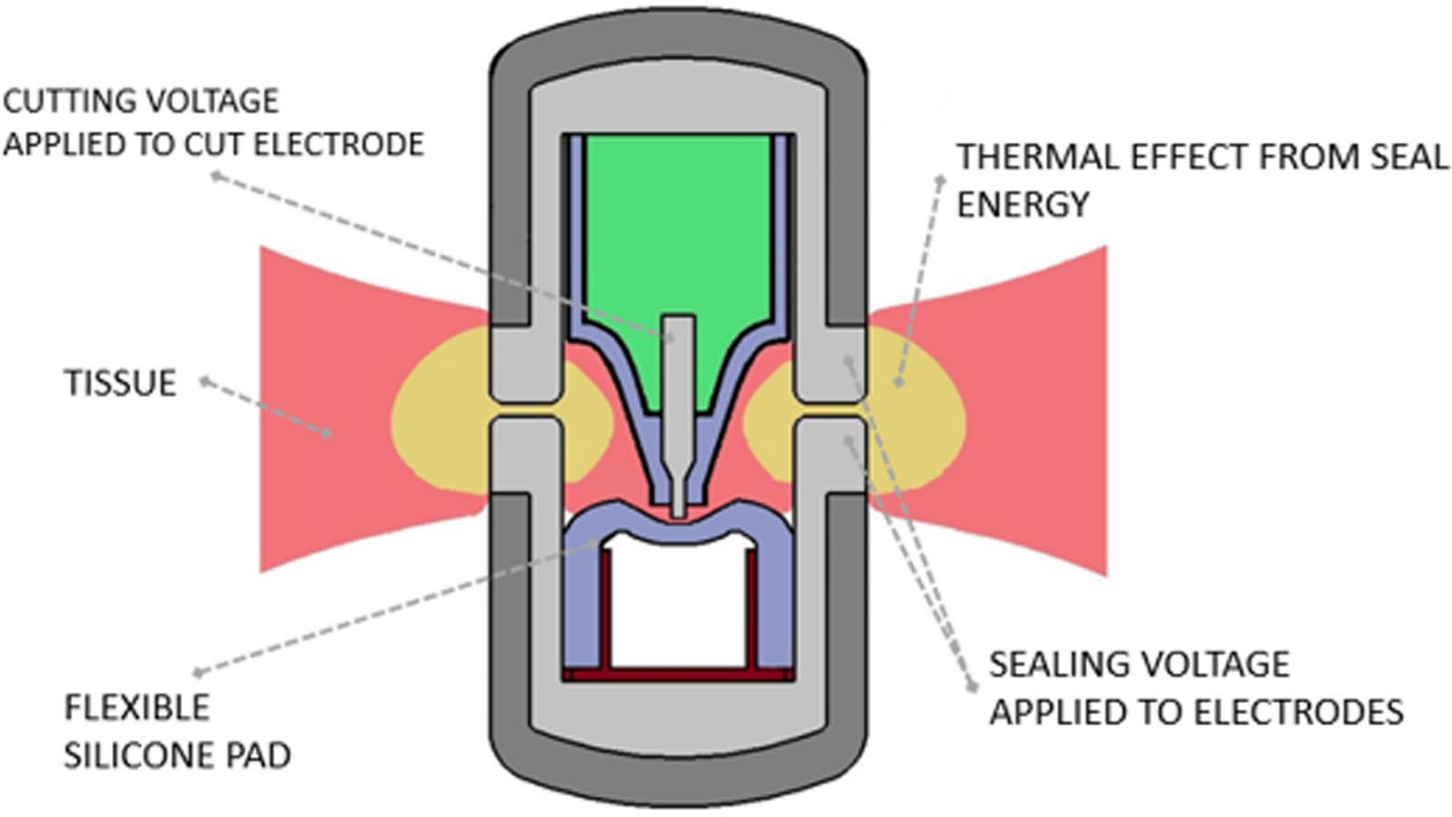
Cross-sectional schematic of tissue clamped in the SynchroSeal jaws

SynchroSeal was designed as a multifunctional, single-use device to increase surgical efficiency by rapidly sealing and transecting tissue and blood vessels while providing grasping and dissection capabilities. It seals and transects simultaneously in a single step and is designed with a slim (2.9-mm), curved profile to facilitate dissection and grasping precision. SynchroSeal applications are based on bipolar electrical RF energy, which is delivered by the E-100 electrosurgical generator within the da Vinci Surgical System, to seal and transect vessels that are ≤5 mm in diameter and tissue bundles that fit within the jaws of the instrument. The surgeon has fully wristed control throughout the process. The two proprietary modes of action are: (1) simultaneous tissue sealing and transection via the “Sync” mode and (2) sealing without transection via the “Seal” mode. In the Sync mode, the raised electrode in one of the jaws applies pressure against a flexible silicone pad, which is seated in the opposing jaw. The sealing surfaces of both jaws apply bipolar seal energy while the cutting electrode independently applies RF energy based on advanced algorithms within the generator that sense the tissue condition (Fig. 1) [7]. Upon activation of the Sync or Seal modes, the jaws increase clamping pressure on the target tissue to ensure adequate compression for successful sealing and transection. The generator monitors the tissue condition and stops delivery of energy once it detects a successful seal and then informs the surgeon with both user interface messages and tones.

Each individual tool—VSE and SynchroSeal—has a unique identification to differentiate for technical data collection. When an adverse event (AE) occurs, the hospital reports it to the manufacturer according to procedure type, date and time of event, device and system used, and surgeon’s name. Data are not connected or correlated to a specific patient. Consequently, Institutional Review Board approval was not required for this effort.

### Bench and ex vivo studies

Grasping capabilities of SynchroSeal and Harmonic Ace+7 were assessed by evaluating the slip resistance force and grip pressure during 12 trials for each device. The ability to grasp tissues with enough strength to prevent tissue from slipping from the instrument jaws while applying low grip pressure to maintain atraumatic control was measured. For slip resistance force, both instruments gripped a uniform material (Tyvek®, Dupont Co.™, Richmond, VA USA) while retraction of the material from the gripped jaws of the instruments was measured to determine the peak slip resistance forces for each device. For grip pressure, the jaws of each device were closed on a Tekscan® pressure sensor (Tekscan, Inc., South Boston, MA), and the grip forces were divided by the contact areas to calculate pressures applied by the instruments.

Sealing times of SynchroSeal with the E-100 generator and Harmonic Ace+7 Shears with Advanced Hemostasis were compared to measure the sealing efficiency of each device. The instruments were used to seal ex vivo porcine renal arteries (Yosemite Meats, Modesto, CA USA) of up to 5 mm in diameter. Both instruments automatically stop energy delivery and provide a tone to the user when the seal cycle is complete. Therefore, seal cycle time was considered as the period from the start of sealing to the seal cycle end tone. Analysis of covariance was used to isolate the effects of the devices on sealing time from the effects of vessel size. Larger vessels generally require longer sealing times because there is more tissue that requires more power delivery [8]. Since vessel size is variable within and between groups, analysis of covariance was performed on sealing times with vessel diameter identified as a covariate and device type identified as a factor.

A lower peak jaw temperature allows for potentially safer vessel sealing as it reduces the risk of unintentional thermal injury [9]. Faster cooling also increases efficiency as it allows the surgeon to move more quickly to the next task while reducing risk of injury to tissue. Temperature profiles of the outside of the jaws of the SynchroSeal and Harmonic Ace+7 devices were measured on ex vivo porcine small bowel mesentery (Yosemite Meats, Modesto, CA USA). Thermal videos were collected during 6 activations of each of the instruments using a thermal camera and the videos were analyzed.

Seal strength of vessels with SynchroSeal was evaluated using burst pressure testing, which involved pressurizing the sealed vessels with deionized water until failure. Ex vivo porcine renal arteries (Yosemite Meats, Modesto, CA USA) with diameters of up to 5 mm were used, with a total of 64 vessels tested for each of the Seal and Sync modes. Mean burst pressure was measured using a technique similar to the one used by Newcomb et al. [8]. A Cole-Parmer® automated injection system (Cole-Parmer, Inc., Vernon Hills, IL USA) with a digital pressure monitor was used to inject deionized water into the lumen of the vessels at a constant rate. The maximum recorded pressure immediately prior to failure was recorded as the burst pressure.

### In vivo analyses

Live porcine animal models (Pork Power Farms, Turlock, CA USA) were used to evaluate thermal spread, seal, and transection performance of SynchroSeal with the E-100 generator. The experiments were approved by an Animal Care and Use Committee. Vessels up to 5 mm in diameter and tissue bundles (set of vessels and/or lymphatic channels that run parallel to each other and are embedded in connective tissue) that fit within the instrument jaws were studied in both Sync and Seal modes. The ability of sealed vessels and the vessels within the tissue bundles to maintain seal integrity intra-operatively was evaluated in live porcine models. Seals were inspected for any signs of leaking, vessel wall compromise (trauma, hole, weakening), or other failure following transection.

A chronic animal study was also performed to assess the long-term outcomes after robotically assisted seal and transection of vessels and tissue bundles in seven porcine hysterectomy and splenectomy procedures with SynchroSeal and the E-100 generator. Either a single vessel (e.g., lineic artery, lineic vein) or a tissue artery and vein bundle (e.g., left gastro-epiploic bundle, short gastric bundle) underwent sealing and transection. The animals were observed for a minimum survival period of 3 weeks, as required by the US Food and Drug Administration to demonstrate safety of vessel sealing instruments [10]. One hundred two vessels were sealed during the procedures. Intraoperative seal performance was assessed immediately after sealing and at the end of the procedure for hemostasis and signs of weakening. During the post-procedure period, the animals were inspected for hemostasis, infection, or unexpected adjacent tissue damage. At 3 weeks, the animals were euthanized while under deep anesthesia via intravenous administration of one to four doses of 40 mEq of potassium chloride, per standard practice, and the sealed sites were excised and sent for histopathology assessment of the healing response.

### Histology

Histopathology evaluation was performed by an independent and blinded pathologist (Alizée Pathology, Inc., Thurmont, MD USA), with the objective of evaluating thermal spread and healing response in the vessels sealed with SynchroSeal.

### Sample size calculations

Pilot testing for peak slip resistance force was performed with SynchroSeal and Harmonic Ace+7 devices. Each device was evaluated 8 times for peak slip resistance force. An a priori Power Test for a two-sample t Test was performed on the pilot data’s difference of 8.25 N, the more conservative standard deviation of 2.65 N, and a power value of 0.8. Based on the pilot data, the power test indicated a required sample size of 3. Due to limitations of the pilot study, including small sample sizes and a planned analysis using Mann-Whitney instead of two-sample t Test, a sample size of 10 was chosen for the current study.

Pilot testing for grip pressure was performed, and each device was tested 12 times. An a priori Power Test for a 2-sample t Test was performed on the pilot data and the sample size for the current study was required to have a power of 0.8. Based on the pilot data, the power test indicated a required sample size of 8. Due to limitations of the pilot study, including small sample sizes and a planned analysis using Mann–Whitney instead of two-sample t Test, a sample size of 12 was chosen to ensure adequate power for the current study.

### Statistical analysis

Descriptive statistics (mean and standard deviation) were used to analyze the data. Mann–Whitney tests were performed to compare Harmonic Ace+7 and SynchroSeal in terms of slip resistance force and grip pressure, peak temperatures and cool down times and to compare SynchroSeal with VSE in the bariatric analyses. In all comparative analyses, statistical significance was set at p < 0.05.

## Results

**Fig. 2:**
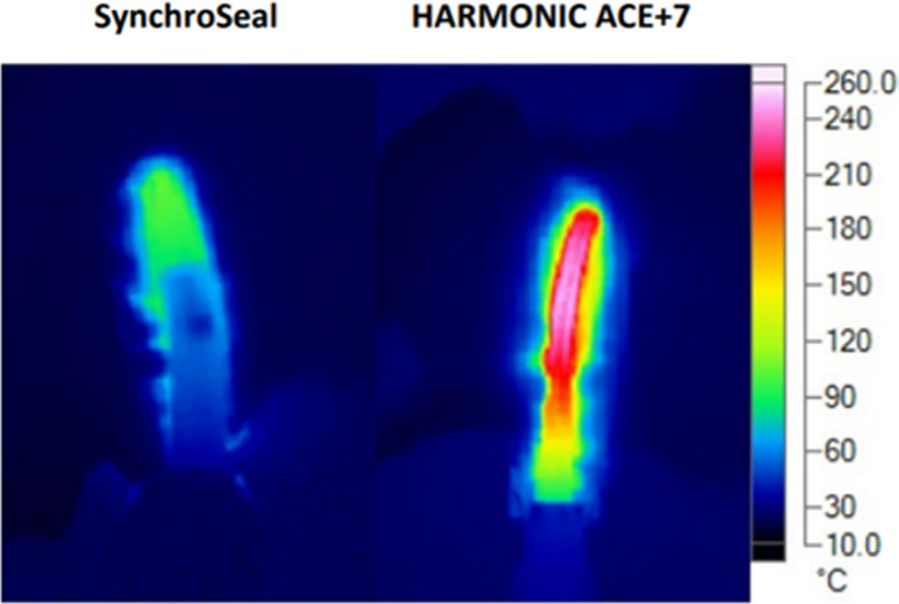
Thermal images of (left) SynchroSeal and (right) Harmonic Ace+7 jaws during peak activation

**Fig. 3:**
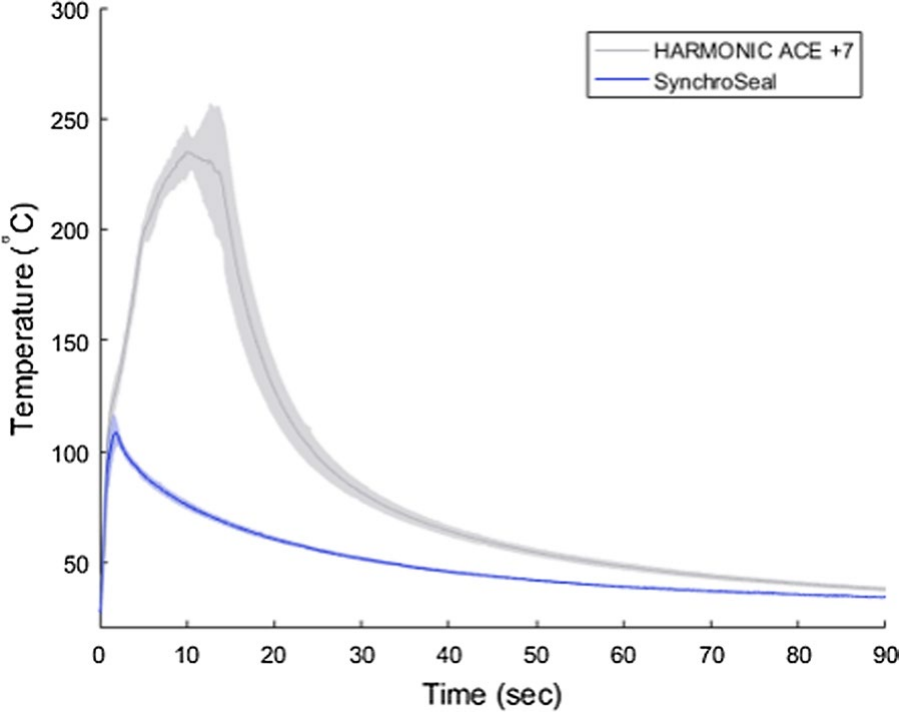
Graphical representation of SynchroSeal and Harmonic Ace+7 jaw temperatures throughout activation and cool down

The results for the bench and ex vivo evaluations of SynchroSeal and Harmonic Ace+7 are tabulated (Table 1) and show the significant differences in test outcomes between the two devices. The slip resistance force of SynchroSeal was higher than that of Harmonic Ace+7 and the grip pressure was lower, indicating SynchroSeal’s stronger and less traumatic grasp. The times required for seal and transection are also presented and evidence a 9.9-second shorter seal time with SynchroSeal. Both instruments sealed and transected vessels in a single activation. Figure 2 shows a thermal image of the lower peak jaw temperature of SynchroSeal and Fig. 3 illustrates its faster cooldown time. The lower peak jaw temperature and faster cooldown may contribute to the safety and efficiency profile of SynchroSeal.

**Table 1:** Bench study evaluations of SynchroSeal and Harmonic Ace+7

Variable	SynchroSeal	Harmonic Ace+7	Mean difference	P-value
Mean slip force, N ± SD	8.4 ± 1.0	3.1 ± 0.4	5.3	0.0002
Mean grip pressure, kg/cm^2^ ± SD	3.0 ± 0.2	4.2 ± 0.5	− 1.2	0.0002
Mean seal time, s ± SD	1.5 ± 0.4	11.6 ± 2.5	− 9.9 s	< 0.0001
Mean peak jaw temperature, °C ± SD	109.7 ± 7.2	247.4 ± 8.6	− 137.7	0.0051
Mean time to cool to 40 °C, s ± SD	53.6 ± 2.1	68.0 ± 3.5	− 14.4	0.0051

*SD* standard deviation of the mean

**Fig. 4:**
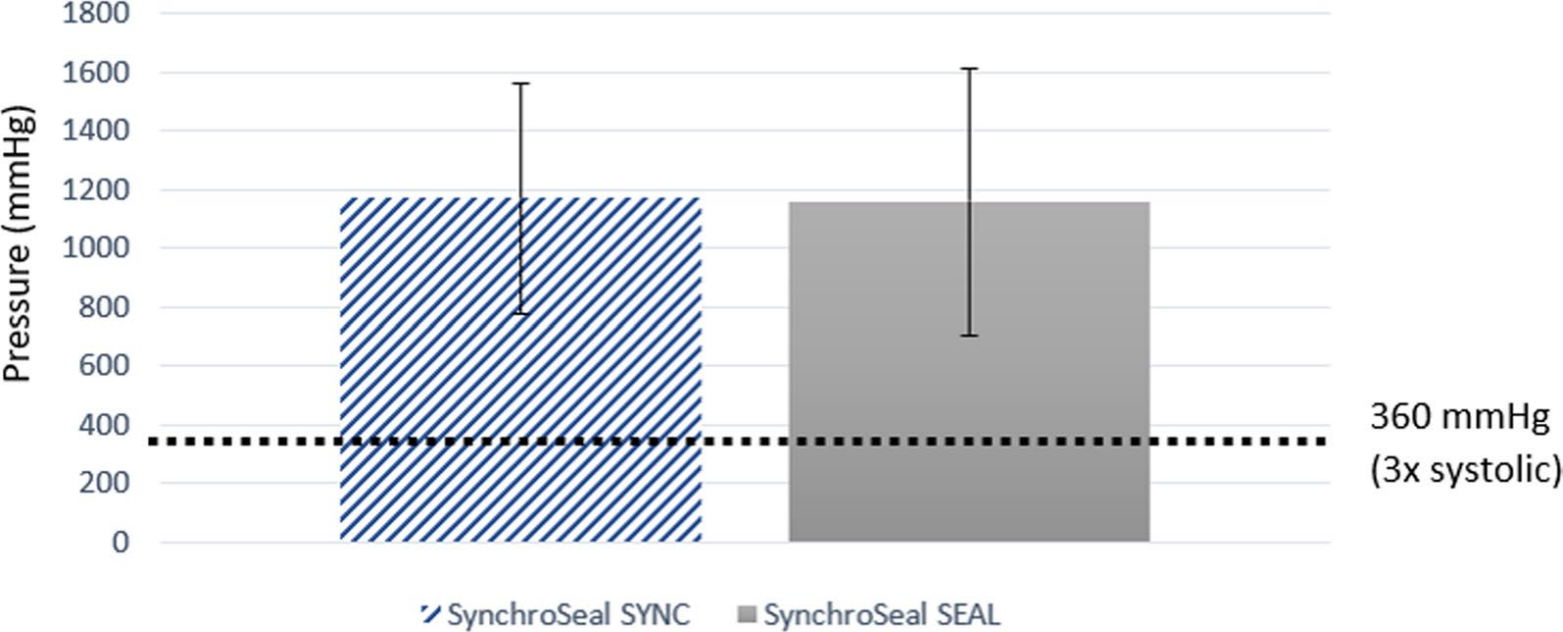
Graphical representation of mean burst pressures under Sync and Seal modes

In the SynchroSeal burst pressure test, sealed arteries evidenced seal integrity at pressures significantly higher than 360 mmHg: three times the normal systolic blood pressure of 120 mmHg. The mean burst pressure in the Sync and in the Seal modes were, respectively, 1169.1 ± 393.1 mmHg and 1159.2 ± 454.6 mmHg (Fig. 4).

**Fig. 5:**
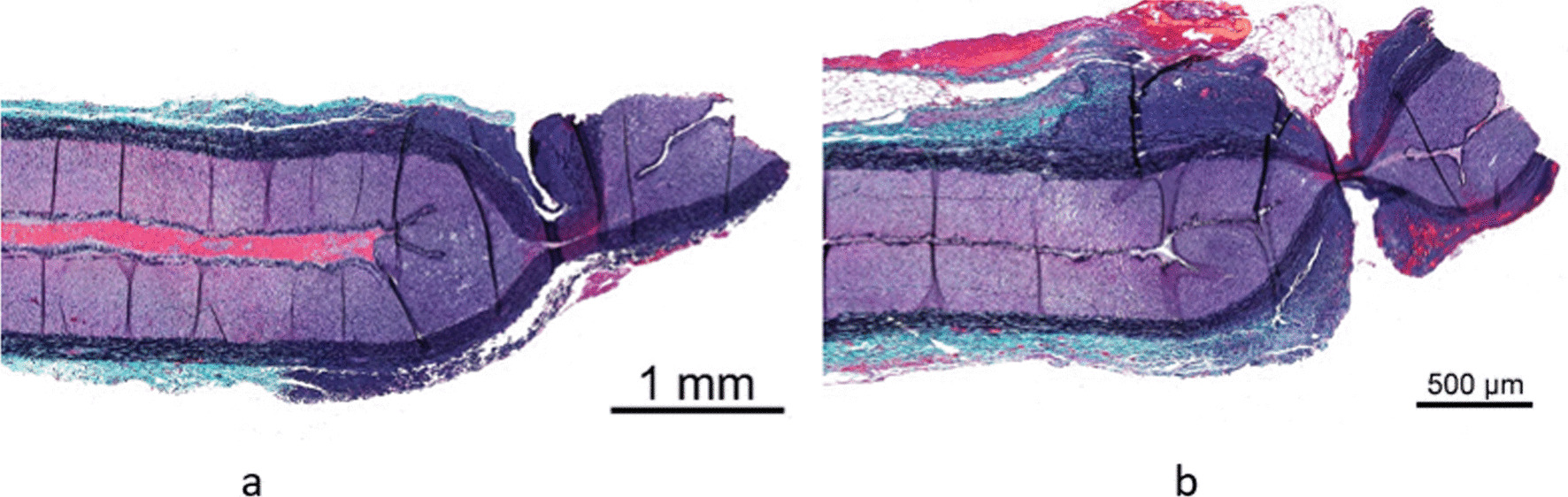
Histology images of an in vivo porcine artery sealed and transected with a single pedal press in **a** Sync mode and **b** Seal mode

All sealed vessels and tissue bundles in the SynchroSeal in vivo porcine study maintained seal integrity intraoperatively. The mean thermal spread with the Sync mode was 1.2 ± 0.6 mm and the mean thermal spread with the Seal mode was 1.5 ± 0.55 mm. A sample of histology images from the study is provided in Fig. 5.

**Fig. 6:**
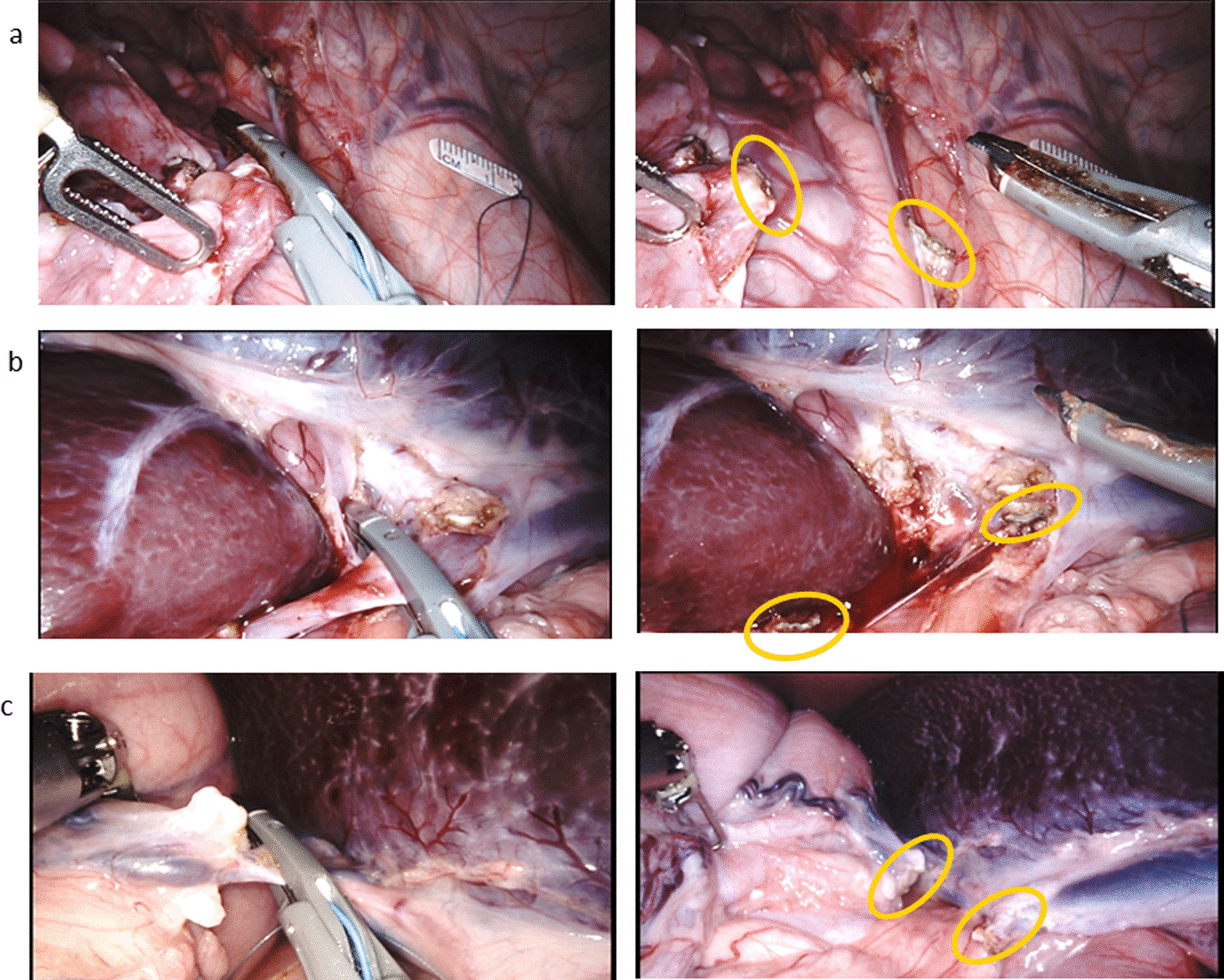
Vessel sealing and transection using SynchroSeal’s Sync mode for **A** ovarian bundle, **B** lineic vein, and **C** short gastric bundle during sealing (left images) and after sealing (right images). Yellow circles show the location of the seals

Images from the hysterectomy and splenectomy procedures in the live porcine models are provided in Fig. 6 and illustrate sealing and transection of vessels and tissue bundles using the Sync mode. Fifty-three seals were made and evidenced no seal failures or signs of weakening and required no additional interventions. Histopathology evaluation of arteries and veins from the splenectomy and hysterectomy at the end of the survival period showed optimal healing with no swelling, discharge, or leakage and no adverse events, such as thermal spread to or damage of non-target tissue.

Sleeve gastrectomy and Roux-en-Y gastric bypass procedures were performed by the senior author (F.R.) at a single medical institution. Procedures that involved use of VSE on ERBE VIO dV were performed from January 1, 2019 to December 1, 2019, and procedures that incorporated SynchroSeal on E-100 were performed from December 5, 2019 to April 15, 2021. Technical data from both procedure groups are provided in Table 2. Regardless of the bariatric procedure, SynchroSeal had a higher number of RF energy activations than VSE during sleeve gastrectomy (79 ± 26 vs. 57 ± 25 activations; *p* < 0.001) and during gastric bypass (74 ± 44 vs. 40 ± 20 activations; *p* < 0.001). However, the mean accumulated RF activation time per procedure for SynchroSeal was less than that for VSE during sleeve gastrectomy (119 ± 38 vs. 249 ± 114 s; *p* < 0.001) and during gastric bypass (108 ± 65 vs. 179 ± 96 s; *p* < 0.001). The use of SynchroSeal as a proportion of the time for all tools used per procedure was more than that of VSE during sleeve gastrectomy (43 ± 17% vs. 35 ± 13%; *p* = 0.007) and gastric bypass (34 ± 18% vs. 20 ± 13%; *p* < 0.001) suggesting its increased multifunctionality by performing more procedure tasks than VSE. The hospital at which the procedures were performed reported no adverse events related to either tool.

**Table 2:** Single center, single user bariatrics analysis comparing Vessel Sealer Extend (Erbe VIO da Vinci generator) and SynchroSeal (E-100 da Vinci generator)

Procedure and variables	Vessel Sealer Extend	SynchroSeal	p-value
Sleeve gastrectomy			
Procedures, n	49	53	
Mean activations per procedure, n ± SD	57 ± 25 ^a^	79 ± 26	< 0.001
Mean accumulated activation time per procedure, s ± SD	249 ± 114 ^a^	119 ± 38	< 0.001
Mean time tool used per procedure, s ± SD	1030 ± 560	1229 ± 445	0.053
Mean tool usage as a proportion of time for all tools used per procedure, % ± SD	35 ± 13	43 ± 17	0.007
Gastric bypass (Roux-en-Y)			
Procedures, n	33	52	
Mean activations per procedure, n ± SD	40 ± 20	74 ± 44	< 0.001
Mean accumulated activation time per procedure, s ± SD	179 ± 96	108 ± 65	< 0.001
Mean time tool used per procedure, s ± SD	934 ± 769	1403 ± 809	0.010
Mean tool usage as a proportion of time for all tools used per procedure, % ± SD	20 ± 13	34 ± 18	< 0.001

*SD* standard deviation of the mean

^a^ Data missing for 4 cases

## Discussion

SynchroSeal is a novel robotic-assisted laparoscopic vessel sealing instrument with RF transection capability that can be used in place of a mechanical knife, enabling simultaneous seal and transection of vessels and tissue bundles. The preclinical testing outcomes of the current study demonstrated that, compared with Harmonic Ace+7, SynchroSeal has superior grasping capabilities, is more efficient in terms of seal activation time and cooldown time, and has a safer profile in terms of jaw temperature. It can successfully seal vessels at pressures significantly higher than the normal systolic pressure, and vessels can maintain their integrity in vivo at the time of the seal and during a post-procedure period of three weeks.

The histology study evidenced a mean thermal spread of less than 2 mm for both Sync and Seal modes, which suggests minimal thermal spread adding to the safety profile of the tool. The mean seal time in pre-clinical testing was quick at 1.5 ± 0.4 s. The narrow, curved jaw profile of SynchroSeal and the rapid Sync mode with the E-100 generator have been designed to enable surgeons to perform surgical tasks difficult to perform with earlier versions of robotic-assisted seal and transection tools. (For example, and in contrast to SynchroSeal, VSE has a straight jaw, incorporates a mechanical blade for tissue transection, and requires multiple pedal presses to seal and transect.) Such tasks include creation of enterotomies and gastrotomies during gastric bypass, dissecting vasculature during colectomy, dissecting the mediastinum and mobilizing the duodenum during esophagectomy, and performing adhesiolysis during ostomy takedown.

In addition to sealing and transection, the multifunctional capabilities of SynchroSeal include grasping, blunt dissection, and tissue access. These are possible due to the grasping force, narrow jaw shape, and wristed control, and may further increase efficiency and surgical flow. The relatively low peak jaw temperature of 109.7 °C with rapid cool down may contribute to the device’s safe profile and low thermal spread as well as to the efficiency of its use with other tools in each procedure. These device attributes may have contributed to SynchroSeal’s use characteristics in a clinical setting by a single user, who was experienced in robotic-assisted bariatric surgery.

### Limitations

Limitations of the current study are its lack of real-world clinical experience with data from multiple surgeons at various stages in their robotic-assisted surgery training and practice. A multicenter clinical study of SynchroSeal is warranted to evaluate its efficacy, safety, and efficiency in clinical settings and across multiple surgical procedures and tasks that might not otherwise involve the use of a single seal-and-transection device. In addition, although the current effort provided detailed preclinical analysis of the device as well as a single user’s clinical commentary, costs were not analyzed. A comprehensive cost effectiveness analysis (which should evaluate direct and indirect costs, including but not limited to SynchroSeal’s multi-functionality that might reduce the need for other dedicated instruments as well as evaluation of its costs in specific procedures in terms of procedure and anesthesia times, lengths of stay, and complications and adverse events with at least 30 days of follow up) was beyond the scope of this technology study. However, costs considerations do warrant further study in future papers.

## Conclusions

Based on bench and ex vivo and in vivo animal study outcomes and outcomes from available clinical data, SynchroSeal’s multifunctional design provides enhanced sealing and transection capabilities with an acceptable safety profile.

## Data Availability

The data that support the findings of this study are available from Intuitive Surgical, Inc., but restrictions apply to the availability of these data, which were used under license for the current study, and so are not publicly available. Data are however available from the authors upon reasonable request and with permission of Intuitive Surgical, Inc.

## Abbreviations

RF: Radiofrequency
VSE: Vessel Sealer Extend
